# Self-reported quality of life, anxiety and depression in individuals with Ehlers-Danlos syndrome (EDS): a questionnaire study

**DOI:** 10.1186/s12891-015-0549-7

**Published:** 2015-04-15

**Authors:** Britta Berglund, Carina Pettersson, Maritta Pigg, Per Kristiansson

**Affiliations:** Department of Public Health and Caring Sciences, Uppsala University, Uppsala, Sweden; Department of Immunology, Genetics and Pathology, Section of Clinical Genetics, Uppsala University Hospital, Uppsala, Sweden

**Keywords:** Connective tissue, Ehlers-Danlos syndrome, SF-36, HADS, Quality of life, Anxiety, Depression, Hypermobility

## Abstract

**Background:**

Many individuals with Ehlers-Danlos Syndrome (EDS) are hypermobile, suffer from long term pain, and have complex health problems. Since these sometimes have no objective physical signs, individuals with EDS sometimes are referred for psychiatric evaluation. The aim was therefore to identify the level of anxiety and quality of life in a Swedish group of individuals with EDS.

**Methods:**

A postal survey in 2008 was distributed to 365 members over 18 years of the Swedish National EDS Association and 250 with EDS diagnosis responded. Two questionnaires, the Hospital Anxiety and Depression Scale (HADS) and SF-36, were used. A Swedish population study was used to compare results from SF-36. Independent Student’s t-test was used to compare differences between groups, possible relationships were tested using Spearman’s correlation coefficient and the General Linear Model was used for regression analyses. Higher scores on HADS represent higher levels of anxiety and depression and higher scores on SF-36 represent higher quality of health.

**Results:**

Of the respondents 74.8% scored high on anxiety and 22.4% scored high on depression on the HADS. Age, tiredness and back pain was independently associated with the HAD anxiety score in a multiple regression analysis, When comparing the SF-36 scores from the EDS group and a Swedish population group, the EDS group scored significantly lower, indicating lower health-related quality of health than the general population (p < 0.001).

**Conclusions:**

In comparison with a Swedish population group, a lower health-related quality of life was found in the EDS group. Also, higher levels of anxiety and depression were detected in individuals with EDS. The importance to explore the factors behind these results and what initiatives can be taken to alleviate the situation for this group is emphasized.

## Background

Ehlers-Danlos syndrome (EDS) is an inherited, lifelong and potentially disabling connective tissue disorder. The prominent features of this disorder are skin laxity, joint hypermobility, chronic joint and limb pain, blood vessel and tissue fragility [1,2]. A phenotypic variance in individuals, from milder to serious presentations with different sets of symptoms, is a problem for the physician when clarifying the diagnosis, such as to clarify the patient’s type of EDS. Six different EDS-types are suggested based on clinical features, genetic and biochemical grounds. The types are called the classical type, the hyper mobile type, the vascular type, the dermatosparaxis type, the arthrochalasia type, and the kyphoscoliotic type [1].

Many aspects of life are affected by the disorder and chronic pain restricts both physical and psychosocial functions [3,4]. When comparing results from Subjective Health Complaints (SHC) in this group with that of the general Norwegian population, the EDS group scored higher than the population on tiredness and chronic pain [4]. The most reported complaints were musculoskeletal and pseudo neurological, followed by gastroenterological complaints [4]. Daily living is complicated by other symptoms as oral problems such as bleeding gums, dental loss, oral pain, difficulty to chew and ineffective anesthetic in treatment [5]. The relation between joint hypermobility syndrome, also known as EDS type III (JHS), and anxiety is studied [6-8]. In comparison with controls, it was found that panic disorder, agoraphobia, and simple phobia were highly associated with JHS. Individuals with hypermobile joints had significantly higher intensity of fears. The researchers thus concluded that JHS and anxiety are related, even in subjects with no psychiatric disorder [7].

In Sweden a number of EDS individuals have joined a patient group, the National EDS Association. Many individuals in the EDS group have complex health problems, are hypermobile and suffer from chronic pain [9]. Since they sometimes don’t have observable physical signs, individuals with EDS often feel humiliated when their problems are neglected by health-care staff [10]. These persons feel invisible in health-care and often struggle for many years to find a physician with whom to discuss their problems. The aim was therefore to identify perceived health-related quality of life and the level of anxiety and depression in a Swedish group of individuals with EDS.

## Methods

### Study population

A postal survey was carried out in 2008 when adult individuals over 18 years (n = 365), all being members of the Swedish National Ehlers-Danlos Syndrome Association, were invited to participate in the study. In the information form was emphasized that responding to the questionnaires was their consent to participate. Results from a randomized Swedish population study were used to compare the SF-36 results from the EDS group.

### Questionnaires

Information was retrieved about age, EDS type, body weight, body height, current cigarette smoking (no/yes), tiredness (no/yes/moderate/severe), current pain presence in the cervical (no/yes), thoracic (no/yes) or sacral regions (no/yes) were retrieved. A back pain variable was created as the sum of cervical, thoracic and sacral regions, ranging from 0 to 3, with 0 meaning no reported back pain.

To measure health related quality of life the Short form Health Survey (SF-36) was used. The SF-36 is a well validated self-rating questionnaire where dimensions of health are assessed, each ranging from 0-100 [11]. Low scores represent low health-related quality of life. With 36 items the following eight dimensions of functioning are described: Physical Functioning, Physical Role Limitation, Bodily Pain, General Health, Vitality, Social Functioning, Emotional Role Limitation, and Mental Health. The reliability and the validity of the Swedish SF-36 version is tested and found satisfactory [12].

The Hospital Anxiety and Depression Scale (HADS) [13] was used to assess levels of anxiety and depression. This self-reported 14-item scale is divided in two dimensions: anxiety (seven items) and depression (seven items). The responses are marked on a Likert scale from zero to four. The responses are summed within dimensions and a total score for each dimension is calculated. The scores can be categorized as follows: 0-7 indicates no anxiety/depression; 8-10 indicates possible anxiety/depression; 11-21 indicates a probable case of anxiety/depression. Higher scores represent higher levels of anxiety and depression. The HADS is found to be sensitive to somatic, psychiatric and primary care patients as well in general population [14].

### Statistical analysis

Summary statistics were computed using standard methods. The Independent Student’s t-test was used to compare differences between groups and the comparison between SF-36 scores was performed between the EDS group and a population group of randomly selected Swedish women and men (n = 8903) (12). Possible relationships based on ordinal data were tested using Spearman’s correlation coefficient. The General Linear Model was used for regression analyses. The scale assigned for categorization of nominal and ordinal factors used in the regression analyses was: female sex (yes/no), current smoking (no/yes), tiredness (0/1/2/3) and back pain (0 to 3). Only two-tailed tests were used. A significance level of 0.05 was considered statistically significant. Statistical analyses were performed using the SAS program package version 9.3 (SAS Institute, Cary, NC).

Ethical approval was given by the Regional Ethics Committee, Stockholm (2008/2:4).

## Results

### Sample

Completed questionnaires were returned by 250 individuals with diagnosis (mean age 46.15; SD 12; range 18-84). 38% of these were individuals with EDS but where the type of EDS not was stated by the physician. The overall response rate was 73%. There were 223 female respondents (89%) and 27 males (11%). A high percentage (80%) reported current smoking. The most frequent EDS variant (38%) was the one where the specific type was not defined, the hypermobility type was as well a common type (30%) (Table 1).

**Table 1:** **Characteristics of the 250 participants with Ehlers-Danlos syndrome (EDS) **

**Characteristic**		**Mean or number (%)**	**C.I**
Age (years)		46.1	44.5 to 47.7
EDS type	Not defined	95 (38)	
	Hypermobility	76 (30)	
	Classic	45 (18)	
	Artrocalasia	2 (1)	
	Vascular	10 (4)	
	Mixed	22 (9)	
Body mass index (kg/m^2^)		25.8	25.1 to 26.5
Current cigarette smoking		200 (80)	
SF-36	Physical component score	27.2	25.8 to 28.6
	Mental component score	42.1	40.6 to 43.6
Tiredness		234 (94)	
Back pain	Cervical region	204 (82)	
	Thoracic region	184 (74)	
	Lumbosacral region	203 (81)	
	Any back region	234 (94)	

CI = 95% confidence interval.

Presented as mean, number and 95% confidence interval.

### Short Form health survey (SF-36)

The dimensions Physical Role Limitation, Bodily pain, General Health and Vitality were rated as the lowest and Mental Health as the highest. The mean Physical component Score (PCS) of 26.0 (CI 24.6 to 27.4) in women and 37.2 (CI 32.4 to 42.0) in men were significantly different (p < 0.0001). The mean Mental component Score (MCS) of 41.5 (CI 39.9 to 43.1) in women and 47.3 (CI 42.3 to 52.4) in men were significantly different (p < 0.03). When comparing the SF-36 scores in the EDS group with the Swedish population group (12), the EDS group reported significantly lower scores, 23.8 to 65 in the EDS group as compared to 68.8 to 88.69 in the population group, indicating lower quality of health than the population group (Table 2).

**Table 2:** **Mean (CI) SF-36 scores in individuals with Ehlers-Danlos’ syndrome (EDS) (n = 250) and a reference group of randomly selected Swedish women and men**

	**EDS respondents n = 250**	**Population group (n = 8903)**	***p*** **T-test**
Physical functioning	48.3 (45.3 to 51.5)	87.9	.000
Physical Role Limitation	23.8 (19.4 to 28.2)	83.2	.000
Bodily Pain	31.6 (29.1 to 34.9)	74.8	.000
General Health	34.2 (31.7 to 37.1)	75.8	.000
Vitality	30.2 (27.8 to 33.0)	68.8	.000
Social Functioning	53.7 (50.4 to 57.4)	88.6	.000
Emotional Role Limitation	58.3 (53.4 to 64.1)	85.7	.000
Mental Health	65.5 (63.0 to 67.9)	80.9	.000

### Hospital Anxiety and Depression Scale (HADS)

The mean anxiety score was 12.0 (CI 11.7 to 12.4) and the mean depression score 9.1 (CI 8.9 to 9.4) (Table 3). The mean anxiety score of 13.1 among men was higher than the score of 11.9 among women (p = 0.26). No difference was shown among the sexes regarding the mean depression scores (p = 0.26). There was a weak negative correlation between anxiety and depression scores (r_s_ = -0.13, p = 0.04 (Table 4). Probable anxiety was rated by 74.8% and probable depression was rated by 22.4% of the participants (Table 4). The items ‘sudden feelings of panic’ and ‘feeling as if something bad will happen’ were rated as the highest of the anxiety measures. ‘I feel cheerful’ and ‘I have lost interest in my appearance’ were rated as the highest in the depression measures (Table 3).

**Table 3:** **The Hospital Anxiety and Depression scale (HADS) with the compounds anxiety and depression**

**Score**		**Mean**	**95 % C.I.**
Anxiety	Total score	12.0	11.7 to 12.4
	I feel tense or wound up	1.9	1.8 to 2.0
	Feeling as if something bad will happen	2.0	1.9 to 2.1
	Worrying thoughts	1.8	1.7 to 2.0
	I can sit at ease and feel relaxed	1.3	1.2 to 1.4
	Feeling like butterflies in the stomach	0.8	0.7 to 0.9
	I feel restless	1.8	1.6 to 1.9
	Sudden feelings of panic	2.4	2.3 to 2.5
Depression	Total score	9.1	8.9 to 9.4
	I enjoy things I used to enjoy	0.8	0.7 to 0.9
	I can laugh and see funny things	0.6	0.5 to 0.7
	I feel cheerful	2.3	2.2 to 2.4
	I feel slowed down	1.6	1.4 to 1.7
	I have lost interest in my appearance	2.2	2.1 to 2.3
	I look forward with enjoyment	1.1	1.0 to 1.2
	I can enjoy a book, radio or TV	0.7	0.6 to 0.8

**Table 4:** **Sum of reported HAD-scores grouped three categories (0-7 no anxiety/depression, 8-10 possible anxiety/depression and 11-21 probable anxiety/depression) among 250 individuals with Ehlers-Danlos’ syndrome**

**Sum of HAD-score**	**Anxiety**	**Depression**
None 0-7	13 (5.2)	46 (18.4)
Possible 8-10	50 (20)	148 (59.2)
Probable 11-21	187 (74.8)	56 (22.4)

Number and proportion is presented.

### Associations between characteristic factors and HAD-score

To find possible factors associated with reported HAD-scores simple and multiple regression analyses were performed. The characteristics of the EDS individuals were used as independent variables and the HAD scores as the dependent variable. In the simple regression analyses, sex and age were positively associated and tiredness and back pain were inversely associated with HAD anxiety score. In the multiple regression analysis age and tiredness were independently associated with HAD anxiety score (Table 5). The R^2^ of the full model was 0.11 (p < 0.0001). No associations between the same characteristics and HAD depression score were shown (data not shown). No collinearity problem was shown (Vif <1.4).

**Table 5:** **Associations between possible determinants and sum of HADS depression and anxiety scores among individuals with Ehlers-Danlos’ syndrome (n = 250)**

	**Simple linear regression**			**Multiple linear regression R** ^**2**^ **= 0.11, p < 0.0001**	
**Variable**	**Estimate**	**R** ^**2**^	**p=**	**Estimate**	**p=**
Sex	0.85	0.01	0.16	0.01	0.98
Age (year)	0.03	0.02	0.03	0.03	0.07
Smoking	0.55	0.00	0.24	0.53	0.23
BMI (kg/m^2^)	-0.07	0.02	0.03	-0.06	0.07
Tiredness	-0.89	0.07	<0.0001	-0.65	0.002
Back pain	-0.54	0.03	0.007	-0.12	0.30

BMI = body mass index.

## Discussion

Among individuals with Ehlers-Danlos syndrome symptoms of probable anxiety and depression were commonly reported as were tiredness and back pain. The estimation of anxiety was three times higher than that of depression. Tiredness and age were measured factors that were independently and significantly associated with anxiety in the EDS group. In addition, the EDS individuals perceived their physical and mental health much lower than a Swedish population group as measured with SF-36.

The findings in this study significantly indicate the perceived lower health-related quality of life in Swedish individuals with EDS as compared with a Swedish population group [12]. Different authors [6,8] have reported that fears and anxiety are related to hypermobility of joints. Findings that have hypermobile joints in focus are interesting since many in the EDS group suffer from the problems that hypermobility causes and strive to find the reason for their health problems. Often these difficulties are neglected by health care due to lack of knowledge of the consequences of hypermobile joints [10].

Women with EDS in the present study reported poorer health-related quality of life than men. This may be related to that women with EDS as well report more subjective health complaints than men with EDS do [4]. In literature, tiredness or fatigue is observed as occurring with pain, unrefreshing sleep and affective symptoms, which may imply a connection between the underlying mechanisms [15]. That women in general report more symptoms than men was also found in a study by Loge and Kaasa [16] in Norway.

This study confirms earlier results that individuals with EDS perceive a lower health-related quality of life than the population. This can be related to different aspects, of which one might be food hypersensitivity, with indigestion, itching and diarrhea as common problems [17]. Individuals with EDS also report probable anxiety and depression. Future research is encouraged to further explore factors behind this and what initiatives can be taken to find treatments to alleviate the situation for this group is stressed.

### Limitations

The major limitation in this study is that data are self-reported due to that the respondents live in all parts of the country. To independently confirm the diagnosis of each person is therefore impossible, however, in the background form a statement was made by each respondent about having received the diagnosis by a physician.

In addition, background variables were not compared to that of the population group,

### Clinical implications

Since probable anxiety and depression and lower health-related quality was found in the present study in this group of individuals with EDS, some implications for health-care can be expected. Individuals in this group may need more personal initiatives from health-care in order to be able to manage daily life. Some suggestions can be to learn about the daily consequences of EDS and to acknowledge the physical and psychosocial differences. It is imperative to be aware of that this is a lifelong, genetic disease with no current treatment needs an individual patient approach.

## Conclusions

A considerably lower health-related quality of life was found among EDS middle-aged individuals, in comparison with a Swedish population group. Also, probable anxiety and depression were detected in individuals with EDS. The importance to explore the factors behind these results in research and what initiatives can be taken to alleviate the situation for this group is emphasized.

## References

[1] Beighton P, De Paepe A, Steinmann B, Tsipouras P, Wenstrup RJ (1998). Ehlers-Danlos syndromes: revised nosology, Villefranche, 1997. Ehlers-Danlos National Foundation (USA) and Ehlers-Danlos Support Group (UK). Am J Med Genet.

[2] Steinmann B, Royce PM, Superti-Furga A, Steinmann B, Royce PM (1993). The Ehlers-Danlos syndrome. Connective tissue and its heritable disorders: molecular, genetic and medical aspects.

[3] Berglund B, Nordström G (2001). Symptoms and functional health status of individuals with Ehlers-Danlos syndrome (EDS). J Clin Rheumatol.

[4] Maeland S, Assmus J, Berglund B (2011). Subjective health complaints in individuals with Ehlers-Danlos syndrome: a questionnaire study. Int J Nurs Stud.

[5] Berglund B, Björck E (2012). Women with Ehlers-Danlos syndrome (EDS) experience low oral health-related quality of life. J Orofac Pain.

[6] Bulbena A, Gago J, Sperry L, Berge D (2006). The relationship between frequency and intensity of fears and a collagen condition. Depress Anxiety.

[7] Bulbena A, Gago J, Pailhez G, Sperry L, Fullana MA, Vilarroya O (2011). Joint hypermobility syndrome is a risk factor trait for anxiety disorders: a 15-year follow-up cohort study. Gen Hosp Psychiatry.

[8] Smith TO, Easton V, Bacon H, Jerman E, Armon K, Poland F (2014). The relationship between benign jointhypermobility syndrome and psychological distress: a systematic review and meta-analysis. Rheumatology (Oxford).

[9] Lumley MA, Jordan M, Rubenstein R, Tsipouras P, Evans MI (1994). Psychosocial functioning in the Ehlers-Danlos syndrome. Am J Med Genet.

[10] Berglund B, Mattiasson AC, Randers I (2010). Dignity not fully upheld when seeking health care: experiences expressed by individuals suffering from Ehlers-Danlos syndrome. Disabil Rehabil.

[11] Ware JE, Kosinski M (2001). Interpreting SF-36 Summary Health Measure: a response. Q Life Res.

[12] Sullivan M, Karlsson J, Ware JE (1995). The Swedish SF-36 Health Survey–I. Evaluation of data quality, scaling assumptions, reliability and construct validity across general populations in Sweden. Soc Sci Med.

[13] Zigmond AS, Snaith RP (1983). The hospital anxiety and depression scale. Acta Psychiatr Scand.

[14] Bjelland I, Dahl AA, Tangen Haug T, Neckelmann D (2002). The validity of the Hospital Anxiety and Depression Scale. An updated literature review. Psychosom Res.

[15] Vincent A, Benzo RP, Whipple MO, McAllister SJ, Erwin PJ, Saligan LN (2013). Beyond pain in fibromyalgia: insights into the symptom of fatigue. Arthritis Research & Therapy.

[16] Loge JH, Kaasa S (1998). Short form 36 (SF-36) health survey: normative data from the general Norwegian population. Scand J Soc Med.

[17] Berglund B: Food hypersensitivity in individuals with EDS: a common problem. Nordic Journal of Nursing Research (accepted 2014).

